# UBASH3A Interacts with PTPN22 to Regulate *IL2* Expression and Risk for Type 1 Diabetes

**DOI:** 10.3390/ijms24108671

**Published:** 2023-05-12

**Authors:** Jeremy R. B. Newman, Patrick Concannon, Yan Ge

**Affiliations:** 1 Department of Molecular Genetics & Microbiology, University of Florida, Gainesville, FL 32610, USA; jrbnewman@ufl.edu; 2 Genetics Institute, University of Florida, Gainesville, FL 32610, USA; patcon@ufl.edu; 3 Department of Pathology, Immunology and Laboratory Medicine, University of Florida, Gainesville, FL 32610, USA

**Keywords:** UBASH3A, PTPN22, IL-2, type 1 diabetes, autoimmunity, rs11203203, rs2476601, T cells

## Abstract

UBASH3A is a negative regulator of T cell activation and IL-2 production and plays key roles in autoimmunity. Although previous studies revealed the individual effects of UBASH3A on risk for type 1 diabetes (T1D; a common autoimmune disease), the relationship of UBASH3A with other T1D risk factors remains largely unknown. Given that another well-known T1D risk factor, PTPN22, also inhibits T cell activation and IL-2 production, we investigated the relationship between UBASH3A and PTPN22. We found that UBASH3A, via its Src homology 3 (SH3) domain, physically interacts with PTPN22 in T cells, and that this interaction is not altered by the T1D risk coding variant rs2476601 in *PTPN22*. Furthermore, our analysis of RNA-seq data from T1D cases showed that the amounts of *UBASH3A* and *PTPN22* transcripts exert a cooperative effect on *IL2* expression in human primary CD8^+^ T cells. Finally, our genetic association analyses revealed that two independent T1D risk variants, rs11203203 in *UBASH3A* and rs2476601 in *PTPN22*, interact statistically, jointly affecting risk for T1D. In summary, our study reveals novel interactions, both biochemical and statistical, between two independent T1D risk loci, and suggests how these interactions may affect T cell function and increase risk for T1D.

## 1. Introduction

Genome-wide association studies and functional studies showed that UBASH3A and its genetic variants contribute to at least five different autoimmune diseases, including type 1 diabetes (T1D) [1,2,3,4,5,6,7,8,9,10,11,12], suggesting a broad role of UBASH3A in autoimmunity. *UBASH3A* is located on human chromosome 21q22.3 and on mouse chromosome 17qA3.3-qB1. *UBASH3A* is expressed primarily in T cells of the adaptive immune system [13,14,15,16].

UBASH3A has three structural domains: the N-terminal ubiquitin-associated (UBA), Src homology 3 (SH3), and the C-terminal histidine phosphatase (also referred to as phosphoglycerate mutase-like [PGM]) domains [13]. The UBA domain can bind to monoubiquitin as well as lysine 63- and methionine 1-linked polyubiquitin chains [16,17]. The SH3 domain interacts with proline-rich domains of other proteins [18,19,20]. The PGM domain mediates self-dimerization [13]; despite the similarity of this domain to phosphatases, UBASH3A exhibits only very weak, possibly acid-dependent phosphatase activity [21,22].

UBASH3A inhibits T cell activation and function [9,14,15,16,21,23,24,25]. We previously showed that UBASH3A downregulates T cell receptor (TCR)-induced NF-κB signaling and thereby suppresses *IL2* expression, a target gene of NF-κB [16]. In addition, UBASH3A regulates the synthesis and dynamics of TCR–CD3 complexes, resulting in attenuated TCR-mediated signaling and reduced IL-2 production [25]. These effects are mediated by the UBA and SH3 domains of UBASH3A, demonstrating phosphatase-independent functions of UBASH3A in T cells [16,25]. We further showed that two genetic variants in *UBASH3A* that are associated with risk of T1D act by enhancing *UBASH3A* expression in human primary T cells upon TCR stimulation, leading to diminished IL-2 production [16].

Although previous studies elucidated the individual effects of UBASH3A and its genetic variants on T1D risk, the relationship of UBASH3A with other T1D risk factors has not been well characterized. To address this issue and gain new insights into the molecular networks underlying autoimmunity, we investigated the interaction of UBASH3A with PTPN22 (located on human chromosome 1p13.2 and on mouse chromosome 3qF2.2), another well-established risk factor for multiple distinct autoimmune diseases including T1D [26,27,28]. The reasons that we focused on PTPN22 are: (1) PTPN22 has four proline-rich motifs (P1-P4) [29], which might bind the SH3 domain of UBASH3A. (2) Similar to UBASH3A, PTPN22 also inhibits T cell activation and IL-2 production [27,28,30,31], but by a different mechanism—PTPN22 acts as a non-receptor tyrosine phosphatase, and it dampens proximal TCR-mediated signaling events by removing activating phosphate residues on key signaling proteins, such as TCR/CD3ζ, LCK, ZAP70 and VAV1 [27,32]. (3) UBASH3A and PTPN22 share common substrates involved in TCR-mediated signal transduction, such as TCR–CD3 complex and ZAP70 [25,27,32]. (4) Both UBASH3A and PTPN22 are risk factors shared by multiple autoimmune diseases, playing crucial roles in autoimmunity [1,2,3,4,5,6,7,8,9,10,11,12,26,27,28].

In this study, we uncover the novel interactions of UBASH3A with PTPN22. Our findings reveal a previously unknown connection between two key risk factors shared by multiple autoimmune diseases, and elucidate the joint action and interplay of these risk factors.

## 2. Results

### 2.1. UBASH3A, via Its SH3 Domain, Interacts with PTPN22 in T Cells

**Figure 1:**
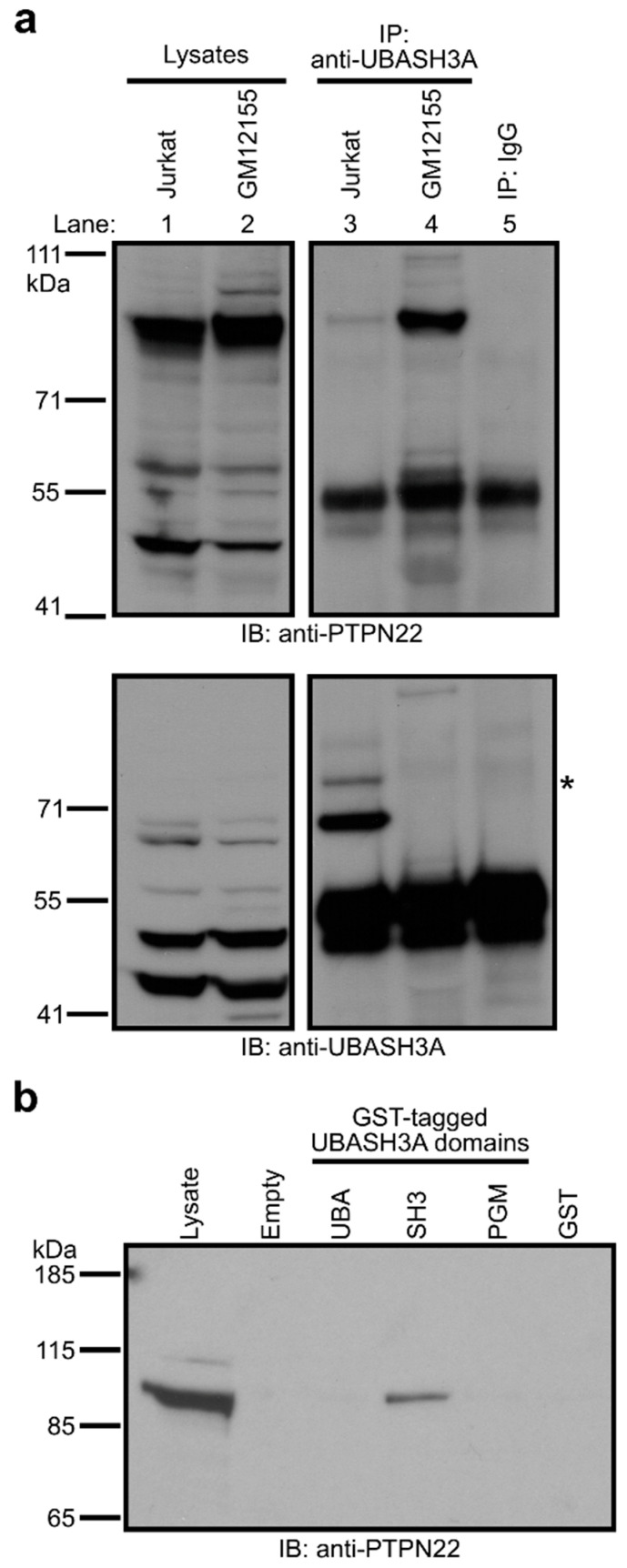
The Src homology 3 (SH3) domain of UBASH3A interacts with PTPN22 in Jurkat cells. (**a**) Lysates from Jurkat cells and GM12155 cells were immunoprecipitated with anti-UBASH3A or IgG. The immunoprecipitates and the input lysates were subjected to immunoblotting with anti-PTPN22, and subsequently, with anti-UBASH3A after stripping. The asterisk indicates the monoubiquitinated form of UBASH3A. The vertical white line indicates that the lanes are not consecutive, although they are of the same gel. Note that the 91 kDa band observed in lane 4 (upper panel) is likely a false positive result because UBASH3A is not detected in GM12155 cells (bottom panel). (**b**) Glutathione S-transferase (GST) pull-down assay using GST or GST-tagged ubiquitin-associated (UBA), SH3, and phosphoglycerate mutase-like (PGM) domains of UBASH3A with lysate from Jurkat cells. The pull-down products and the Jurkat lysate were subjected to immunoblotting with anti-PTPN22.

To determine whether UBASH3A interacts with PTPN22 at the protein level, we performed co-immunoprecipitation using lysates from Jurkat cells (a human T cell line) and GM12155 (a human B-lymphoblastoid cell line). Our results show that Jurkat cells expressed UBASH3A and its monoubiquitinated form (Figure 1a, bottom panel), and that UBASH3A co-immunoprecipitated with PTPN22 in Jurkat cells (Figure 1a, top panel). UBASH3A was not detected in GM12155 cells (Figure 1a, bottom panel), which is consistent with the previous report that murine Ubash3a protein was detected in splenic T cells, but not in splenic B cells [14].

**Figure 2:**
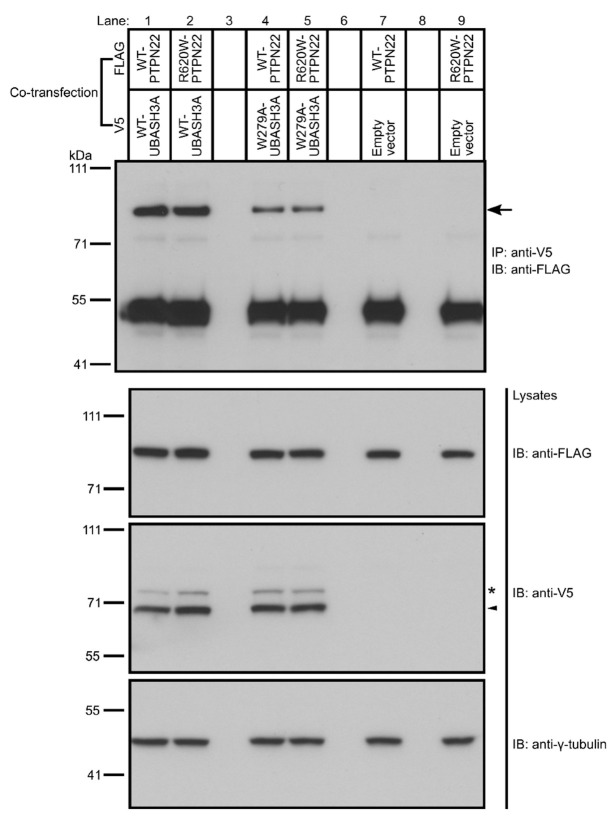
Effects of the UBASH3A W279A mutation (in the SH3 domain) and the single-nucleotide polymorphism (SNP) rs2476601 (in *PTPN22*) on the interaction between UBASH3A and PTPN22. HEK293T cells were co-transfected with constructs encoding the indicated form of FLAG-tagged PTPN22 (wild-type [WT] or R620W [the missense change caused by the minor, risk allele at rs2476601]) and the indicated form of V5-tagged UBASH3A (wild-type [WT] or W279A [SH3 mutant]) or V5. Lysates from the transfected cells were immunoprecipitated with anti-V5. The immunoprecipitates were subjected to immunoblotting with anti-FLAG. The same lysates were subjected to immunoblotting with anti-FLAG, and subsequently, with anti-V5 and anti-γ-tubulin after stripping. The arrow indicates FLAG-tagged wild-type or R620W PTPN22. The arrowhead indicates V5-tagged UBASH3A. The asterisk indicates the monoubiquitinated form of V5-tagged UBASH3A.

To fine map the interaction of UBASH3A with PTPN22, we carried out glutathione S-transferase (GST) pull-down assays using cell lysates from Jurkat cells and GST-tagged UBA, SH3 and PGM domains of UBASH3A, or GST (as a negative control). Our data show that only the SH3 domain of UBASH3A pulled down PTPN22 (Figure 1b). To confirm this finding, we examined whether the W279A mutation in the SH3 domain of UBASH3A affects its binding to PTPN22. As shown previously, the W279A mutation of UBASH3A mitigates the function of its SH3 domain and disrupts the binding of UBASH3A to CBL [19] and CBL-B [25]. We transfected HEK293T cells with a construct encoding FLAG-tagged wild-type PTPN22 together with another construct encoding V5-tagged wild-type or W279A UBASH3A. Lysates from the transfected HEK293T cells were subjected to co-immunoprecipitation and immunoblotting analysis. Our data show that the W279A mutation resulted in a reduction in the binding of UBASH3A to PTPN22 (Figure 2, Lane 1 and 4). Together, our data demonstrate that the SH3 domain of UBASH3A mediates its interaction with PTPN22.

### 2.2. rs2476601 Does Not Alter the Interaction between UBASH3A and PTPN22

PTPN22 has four proline-rich motifs (P1-P4) at the C-terminus, and the P1 motif interacts with the SH3 domain of CSK [29,33]; rs2476601 (GRCh38 NC_000001.10:g.114377568A>G) is located in the P1 motif of PTPN22; rs2476601 is associated with more than ten autoimmune diseases, including T1D [26,27,28]. The minor risk allele at rs2476601 results in a change from arginine (R) to tryptophan (W) at amino acid position 620, leading to a reduction in the binding of PTPN22 to CSK [26] and alterations in the functions of immune cells, including T cells [27,28].

To determine whether rs2476601 affects the interaction of UBASH3A with PTPN22, we transfected HEK293T cells with a construct encoding FLAG-tagged wild-type or R620W PTPN22 together with another construct encoding V5-tagged wild-type or W279A UBASH3A. Lysates from the transfected HEK293T cells were subjected to co-immunoprecipitation and immunoblotting analysis. Our results show that the R620W substitution corresponding to the minor risk allele at rs2476601 does not alter the binding of PTPN22 to wild-type UBASH3A (Figure 2, Lane 1 and 2) or to W279A UBASH3A (Figure 2, Lane 4 and 5).

### 2.3. UBASH3A and PTPN22 Transcripts Cooperatively Regulate IL2 Expression in Human Primary CD8^+^ T cells from T1D Cases

Both UBASH3A and PTPN22 inhibit T cell activation and IL-2 production [9,14,15,16,21,23,24,25,27,28,30,31], but by different molecular mechanisms: UBASH3A regulates the synthesis and dynamics of TCR–CD3 complexes and attenuates NF-κB signaling in a ubiquitin-dependent manner [16,25], whereas PTPN22 functions as a phosphatase and downregulates proximal TCR signal transduction [27,28,29,32]. Previously, we showed that in stimulated human primary T cells, *UBASH3A* and *IL2* mRNA levels were inversely correlated [16], two T1D risk genetic variants in *UBASH3A* led to more *UBASH3A* transcripts and less *IL2* transcripts [16], and a T1D-protective variant in *UBASH3A* led to less *UBASH3A* transcripts and more *IL2* transcripts [9]. These prior findings and our new observation of the binding of UBASH3A to PTPN22 prompted us to test the hypothesis that *UBASH3A* and *PTPN22* transcripts cooperatively regulate *IL2* expression in T cells. We analyzed RNA-seq data from primary CD4^+^ T and CD8^+^ T cells that were purified from peripheral blood samples from 80 T1D cases [34]. Our linear mixed-effects models revealed that the interaction term of *UBASH3A* and *PTPN22* mRNA levels had a statistically significant impact on *IL2* mRNA level in CD8^+^ T cells from T1D cases (*p* = 0.0009, Table 1), but not in CD4^+^ T cells (*p* = 0.62). Our results indicate that the amounts of *UBASH3A* and *PTPN22* transcripts exert a cooperative effect on *IL2* transcript levels in resting CD8^+^ T but not CD4^+^ T cells from T1D cases.

### 2.4. Two Independent T1D Risk Genetic Variants in UBASH3A and PTPN22 Jointly Affect Risk for T1D

It was shown that rs11203203 (GRCh38 NC_000021.9:g.42416077G>A) in the *UBASH3A* gene is a credible causative variant for T1D [8], and rs11203203 is located in a potential enhancer in intron 4 of *UBASH3A* [8]. Previously, we demonstrated that in human primary T cells upon TCR stimulation, the minor risk allele at rs11203203 results in enhanced *UBASH3A* expression and diminished *IL2* expression [16]. Although prior studies from our group and others elucidated the individual effects of single-nucleotide polymorphisms (SNPs) in *UBASH3A* and *PTPN22* on risk for T1D [9,16,26], the joint action of those SNPs in two independent T1D risk loci in disease development has not been well characterized.

Given our observation of a biochemical interaction between UBASH3A and PTPN22 and functional effects related to their transcript levels, we hypothesized that genetic interaction between these two risk loci contributes to T1D. To test this hypothesis, we examined the joint effect of rs11203203 (in *UBASH3A*) and rs2476601 (in *PTPN22*) on risk for T1D by performing genetic association analyses using two different programs, UNPHASED [35] and MDR-PDT [36]. We used data from 10796 individuals from 2689 T1D-affected sibling pairs and trio families of European ancestry ascertained by the Type 1 Diabetes Genetics Consortium (T1DGC) [37]. Our UNPHASED analysis revealed that the interaction between rs11203203 and rs2476601 had a statistically significant impact on risk for T1D (*p* = 0.029). This finding was confirmed by our 2-SNP analysis using MDR-PDT (*p* = 0.001, Table 2). Together, these data indicate that independent genetic variants in *UBASH3A* and *PTPN22* statistically interact, jointly affecting risk for T1D.

## 3. Discussion

This study uncovers novel interactions between two independent autoimmune risk loci, UBASH3A and PTPN22, each of which contribute to multiple distinct autoimmune diseases [1,2,3,4,5,6,7,8,9,10,11,12,26,27,28]. We identified and characterized a novel biochemical interaction between UBASH3A and PTPN22 in T cells, and showed that this interaction is mediated by the SH3 domain of UBASH3A, likely via interaction with one of the four proline-rich motifs of PTPN22. Further studies will be required to determine whether a single or multiple proline-rich motifs in PTPN22 facilitate this interaction. We also observed a statistical interaction between the risk variants at *UBASH3A* and *PTPN22* that is distinct from the physical interaction of these proteins, as evidenced by the lack of impact of the rs2476601 coding variant on the co-immunoprecipitation of UBASH3A and PTPN22 (Figure 2).

In addition, we analyzed our T1D RNA-seq dataset profiling the transcriptomes of primary CD4^+^ T and CD8^+^ T cells (without *in vitro* stimulation) from T1D patients [34]. Our results show that in T1D CD8^+^ T cells, there was a statistical interaction between *UBASH3A* and *PTPN22* transcripts, regulating *IL2* expression; more *UBASH3A* and *PTPN22* transcripts led to an increase in *IL2* expression, as indicated by the positive coefficient estimate for the parameter “*UBASH3A* × *PTPN22* mRNA expression” (Table 1). IL-2 is a crucial cytokine involved in T cell survival, activation, and differentiation [38], and low IL-2 levels were observed in patients with T1D [39,40], systemic lupus erythematosus [41,42], or rheumatoid arthritis [43]. Our findings demonstrate the complexity of IL-2 regulation, and suggest several possible underlying biological mechanisms. One possibility is that higher mRNA levels of *UBASH3A* and *PTPN22* enhance their interaction, resulting in the degradation of PTPN22, given that UBASH3A is involved in the ubiquitin system and protein turnover [16,25], and/or in the suppression of the functions of UBASH3A and PTPN22. Another possibility is that elevated *UBASH3A* and *PTPN22* mRNA levels trigger the expression of other genes that promote *IL2* expression in T cells from T1D patients. These possibilities can be further examined in T cells from healthy subjects and from subjects with autoimmune disease, as well as in mouse models of autoimmune disorders, such as the NOD strain, in which both Ubash3a and Ptpn22 were shown to contribute to the development of T1D [44,45].

Currently, low-dose IL-2 is being tested in clinical trials as a therapy for several autoimmune diseases; however, it remains a challenge to find the optimal effective IL-2 dosage for each individual [46]. Our findings suggest that genetic testing might be useful in dose optimization, particularly in subjects with autoimmunity where the relevant risk alleles at rs2476601 and rs11203203 occur at increased frequencies. In addition, the results of this study may facilitate the development of new combinatorial therapies for autoimmune disease that target both UBASH3A and PTPN22 or the key molecular pathways involving these two risk factors.

## 4. Materials and Methods

### 4.1. Sample Information

Genotype and phenotype data used in this study are available in dbGaP (https://www.ncbi.nlm.nih.gov/gap (accessed on 29 March 2023); Study Accession: phs000911.v1.p1). All biospecimens and data were represented by only non-identifying codes.

### 4.2. Cell Culture and Lysis

Jurkat (clone E6-1; American Type Culture Collection, Manassas, VA, USA) cells and GM12155 (Coriell Institute, Camden, NJ, USA) cells were cultured in RPMI 1640 medium supplemented with 10% heat-inactivated FBS, 2.05 mmol/L L-glutamine, 100 units/mL penicillin, 100 mg/mL streptomycin, and 1 mmol/L sodium pyruvate at 37 °C with 5% CO_2_. HEK293T (American Type Culture Collection, Manassas, VA, USA) cells were cultured in DMEM medium supplemented with 10% FBS, 100 units/mL penicillin, 100 mg/mL streptomycin, and 1 mmol/L sodium pyruvate at 37 °C with 5% CO_2_.

Whole-cell lysates were extracted using EBC lysis buffer (50 mM Tris-HCl, pH 7.5, 120 mM NaCl, 0.5% Nonidet P-40 [NP-40], and 1 mM EDTA) containing protease and phosphatase inhibitors (cOmplete Mini and PhosSTOP; Roche Diagnostics GmbH, Mannheim, Germany) as previously described [47].

### 4.3. Co-Immunoprecipitation and Immunoblotting

Co-immunoprecipitation and immunoblotting were performed as previously described [47]. In brief, 0.3–1 mg of whole-cell lysate was incubated for 4 h or overnight at 4 °C with IgG or the indicated antibodies to human UBASH3A or V5. Next, 20 μL of Protein G Dynabeads (Thermo Fisher Scientific, Lafayette, CO, USA) was added and incubated with the lysate for 1 h at 4 °C. The beads were washed four times with EBC lysis buffer, and the immunoprecipitates were eluted by heating the beads for 10 min at 70 °C in 20 μL of LDS sample buffer (Thermo Fisher Scientific, Lafayette, CO, USA) containing 50 mM DTT. Cell lysates and the immunoprecipitated samples were resolved on NuPAGE Tris-Acetate or Bolt Bis-Tris Plus gels (Thermo Fisher Scientific, Lafayette, CO, USA). After transfer to PVDF membranes, immunoblotting was performed using the indicated antibodies.

The following antibodies were used: mouse anti-FLAG (F1804-1MG, MilliporeSigma, Saint Louis, MO, USA), mouse anti-γ-tubulin (T6557, MilliporeSigma, Saint Louis, MO, USA), goat anti-human PTPN22 (AF3428, R&D Systems, Minneapolis, MN, USA), rabbit anti-human UBASH3A (SAB1410972-100UG, MilliporeSigma, Saint Louis, MO, USA; 15823-1-AP, Proteintech Group, Rosemont, IL, USA), and mouse anti-V5 (R960-25, Thermo Fisher Scientific, Lafayette, CO, USA).

### 4.4. Glutathione S-Transferase Pulldown

Glutathione S-transferase pull-down assays were performed as previously described [16]. In brief, whole-cell lysate from unstimulated Jurkat cells was precleared by incubation with Glutathione Sepharose 4B resin (GE Healthcare Life Sciences, Warrensville Heights, OH, USA) for 2 h at 4 °C. One milligram of the precleared lysate was incubated for 2 h at 4 °C with 20 μL of packed, fresh Glutathione Sepharose 4B resin and with one of the following at the same molar concentration: 11.6 mg of GST-tagged UBA (residues 20–60), 12.6 mg of GST-tagged SH3 (residues 241–300), or 22.9 mg of GST-tagged PGM (residues 316–623) domain of UBASH3A (NCBI Reference Sequence: NP_001001895.1) and 10 mg of GST. After four washes with EBC lysis buffer, eluates were extracted from the resin using LDS sample buffer (Thermo Fisher Scientific, Lafayette, CO, USA) containing 50 mM DTT and analyzed by immunoblotting.

### 4.5. Transfection of HEK293T Cells

As previously described [25], a cDNA of the full-length transcript of *UBASH3A* (CCDS 33566.1) was cloned into the pcDNA3.1/nV5-DEST expression vector (Thermo Fisher Scientific, Lafayette, CO, USA), and a cDNA of the full-length transcript of *PTPN22* (CCDS 863.1) into the pCMV-Tag2 vector (Agilent Technologies, Santa Clara, CA, USA). Site-directed mutagenesis was used to generate the mutant forms of *UBASH3A* and *PTPN22*. HEK293T cells were transfected using the X-tremeGENE HP DNA Transfection Reagent (Roche Diagnostics GmbH, Mannheim, Germany), following the manufacturer’s protocol.

### 4.6. RNA Sequencing

RNA sequencing of CD4^+^ T and CD8^+^ T cells (without in vitro stimulation) from 83 T1D cases was previously described in Newman et al. [34], and the dataset is publicly available in dbGaP (https://www.ncbi.nlm.nih.gov/gap (accessed on 29 March 2023); Accession Number: phs001426.v1.p1). In brief, 83 T1D cases ascertained by the T1DGC were selected for RNA-seq. CD4^+^ T and CD8^+^ T cells were purified from peripheral blood mononuclear cells by positive selection with antibody-coated magnetic beads (Miltenyi Biotec, Auburn, CA, USA). RNA was purified, libraries prepared, and sequencing (50 million reads/sample) performed using the Illumina HiSeq 2000 platform. Samples were prepared and sequenced in three pools with paired-end 50-bp reads. For samples passing quality control [34], paired-end RNA-seq reads were aligned to human Ensembl (Release 99) transcript sequences using Bowtie [48] (version 0.12.9), and transcript quantities were estimated with RSEM [49] (version 1.2.28) with default settings. References for RSEM were prepared with “rsem-prepare-reference”, and transcript sequences for the complete Ensembl transcriptome (release 99) were derived from the GRCh38/hg38 reference genome and a tab delimited gene-to-transcript index file [50]. Upper-quartile normalization and natural log transformation were applied to the coverage data [51,52]. Three subjects with low-coverage RNA-seq samples were removed, and the remaining 80 subjects (42 males and 38 females) were used for subsequent analyses.

### 4.7. Linear Mixed-Effects Models

To assess the joint effect of *UBASH3A* and *PTPN22* transcripts on *IL2* expression, the T1D RNA-seq data for each cell type (CD4^+^ T and CD8^+^ T) were modeled as

*y*_*ijk*_ = *μ* + *a*_*ijk*_ + *b*_*ijk*_ + (*ab*)_*ijk*_ + *s*_*i*_ + *g*_*j*_ + *p*_*k*_ + **V**_*j*_ + *ε*_*ijk*_

where *y_ijk_*, *a_ijk_*, and *b_ijk_* are the log-transformed normalized expression of *IL2*, *UBASH3A*, and *PTPN22*, respectively; *ab* is the interaction between *UBASH3A* and *PTPN22* expression; *s* is sex (*i* = male, female); *g* is age of a subject at ascertainment; *p* is the RNA-seq pool (*k* = 1, 2, 3); **V** is the first three PEER factors, to explain hidden confounders [53]; *j* is the individual subject (*j* = 1, 2, ..., 80). Variables sex (*s*) and PEER factors (**V**) were fixed effects, and pool (*p*) was considered as a random effect distributed ~N(0, σ_k_). The residual ε were assumed to be distributed ~N(0, σ_j_), and degrees of freedom were adjusted using Kenward–Roger approximations [54]. Additional linear mixed-effects modeling was performed and revealed that sex does not affect the expression levels of *UBASH3A* and *PTPN22* in primary CD4^+^ T or CD8^+^ T cells from T1D cases.

### 4.8. Statistical Analyses

Linear mixed-effects modeling was conducted in SAS (version 9.4; SAS Institute, Cary, NC, USA). Mendelian inconsistency in the genotyping dataset was checked with PLINK [55] (version 1.9). Genetic interaction and association analyses were conducted with the UNPHASED [35] (version 3.1.5) and MDR-PDT [36] (version 2.0.1.21) programs. The following flags were used in the UNPHASED analysis: -marker rs11203203 -condition rs2476601 -model gxg -genotype -missing -nolinkage. The default settings of MDR-PDT were used, and the model size was set to 1–2.

## Figures and Tables

**Table 1:** Linear mixed-effects modeling of RNA-seq data from primary CD8^+^ T cells from 80 T1D cases.

Parameter Name ^1^	Coefficient Estimate	Standard Error	Degrees of Freedom	Lower CL ^2^	Upper CL ^3^	T Value	ANOVA F Value	ANOVA *p* Value
*UBASH3A* mRNA expression	−1.2057	0.3575	69	−1.9189	−0.4925	−3.37	11.37	0.0012
*PTPN22* mRNA expression	−1.1718	0.3614	69	−1.8928	−0.4508	−3.24	10.51	0.0018
*UBASH3A* × *PTPN22* mRNA expression	0.3364	0.0965	69	0.1439	0.5288	3.49	12.16	0.0009
Subject sex	−0.0284	0.0317	69	−0.0917	0.0349	−0.89	0.80	0.3745
Subject age	0.0026	0.0019	69	−0.0011	0.0064	1.42	2.02	0.1602
RNA-seq pool	−0.0209 −0.0406	0.0484 0.0468	69 69	−0.1174 −0.1340	0.0756 0.0527	−0.43 −0.87	0.43	0.6530
PEER factor 1	−18.5866	7.7675	69	−34.0823	−3.0910	−2.39	5.73	0.0194
PEER factor 2	3.8907	2.0705	69	−0.2399	8.0213	1.88	3.53	0.0645
PEER factor 3	5.8298	3.7017	69	−1.5549	13.2146	1.57	2.48	0.1199

^1^ Model: log upper quartile-normalized *IL2* mRNA~log upper quartile-normalized *UBASH3A* mRNA + log upper quartile-normalized *PTPN22* mRNA + log upper quartile-normalized *UBASH3A* mRNA * log upper quartile-normalized *PTPN22* mRNA + sex + age + RNA-seq pool + PEER factors (see Section 4 for details). ^2^ Lower bound of the 95% confidence interval for the coefficient estimate. ^3^ Upper bound of the 95% confidence interval for the coefficient estimate.

**Table 2:** MDR-PDT analysis of rs11203203 (in *UBASH3A*) and rs2476601 (in *PTPN22*).

Analysis	Model	T Statistic	Matched Odds Ratio	*p* Value
1-SNP	rs11203203	2.754	1.243	0.992
1-SNP	rs2476601	8.087	2.277	0.001
2-SNP	rs11203203-rs2476601	8.094	2.280	0.001

## Data Availability

Publicly available datasets were analyzed in this study. This data can be found here: https://www.ncbi.nlm.nih.gov/gap (accessed on 29 March 2023); Study Accession: phs000911.v1.p1; https://www.ncbi.nlm.nih.gov/gap (accessed on 29 March 2023); Accession Number: phs001426.v1.p1. The data presented in this study are available in this article.
